# The anti-apoptotic factor Che-1/AATF links transcriptional regulation, cell cycle control, and DNA damage response

**DOI:** 10.1186/1747-1028-2-21

**Published:** 2007-07-16

**Authors:** Claudio Passananti, Maurizio Fanciulli

**Affiliations:** 1 Instituto di Biologia e Patologia Molecolare, CNR, c/o Regina Elena Cancer Institute, Via Delle Messi d'oro 156, 00158 Rome, Italy; 2 Laboratory "B", Department of Therapeutic Programs Development, Regina Elena Cancer Institute, Via Delle Messi d'oro 156, 00158 Rome, Italy; 3 Rome Oncogenomic Center, Regina Elena Cancer Institute, Rome, Italy

## Abstract

Che-1 is a RNA polymerase II binding protein involved in the transcriptional regulation of E2F target genes and in cell proliferation. Recently, it has been shown that Che-1 accumulates in cells responding to genotoxic agents such as Doxorubicin and ionizing radiation. The DNA damage-activated checkpoint kinases ATM and Chk2 interact with and phosphorylate Che-1, enhancing its accumulation and stability, and promoting Che-1-mediated transcription of p53-responsive genes and of p53 itself, as evidenced by microarray analysis. This transcriptional response is suppressed by expression of a Che-1 mutant lacking ATM and Chk2 phosphorylation amino acid residues, or by depletion of Che-1 by RNA silencing. In addition, chromatin immunoprecipitation analysis has shown that Che-1 is released from E2F target genes and recruited to the p21 and p53 promoters after DNA damage. Che-1 contributes to the maintenance of the G_2_/M checkpoint in response to genotoxic stress. These findings identify a new mechanism by which the checkpoint kinases regulate, via the novel effector Che-1, the p53 pathway. Lastly, increasing evidence suggests that Che-1 may be involved in apoptotic signaling in neural tissues. In cortical neurons, Che-1 exhibits anti-apoptotic activity, protecting cells from neuronal damage induced by amyloid β-peptide. In cerebellar granule neurons, Che-1 interacts with Tau in the cytoplasmic compartment and this interaction is modulated during neuronal apoptosis. Finally, Che-1 directly interacts with the neuronal cell-death inducer "NRAGE" which downregulates endogenous Che-1 by targeting it for proteasome-dependent degradation. These findings identify Che-1 as a novel cytoprotective factor against apoptotic insults and suggest that Che-1 may represent a potential target for therapeutic application.

## Background

Che-1, also called AATF or Ded, is a phosphoprotein containing 558 amino acids and has been identified as a RNA polymerase II binding protein in a two-hybrid screen [1]. Human Che-1 was also cloned by differential display of genes downregulated by TGFβ [2]. The human *Che-1 *gene is located at chromosome 17q11.2-q12, and Che-1 is highly conserved among eukaryotic species [2]. Che-1 expression is essential during the early steps of embryogenesis. Indeed, in the absence of a fully functional *Traube *gene (the murine ortholog of Che-1), embryos halt in development at the compacted morula stage and die 2 days later [3].

One essential role of Che-1 is to promote cellular transcription as an adaptor or mediator that links specific transcription factors to the general transcription apparatus. Che-1 per se does not appear to possess DNA binding activity, but Page et al. [4] showed that Che-1 exhibits transactivation activity in a Gal4-linked reporter assay in yeast, although in our hands only the acidic domain at the N terminus of the molecule possesses this transactivation activity. Che-1 binds nuclear hormone receptors directly in vitro and enhances transactivation by several steroid hormone receptors, including androgen, estrogen, and glucocorticoid receptors in a hormone-dependent manner [5]. In addition, Che-1 interacts with the tumor suppressor TSG101, and together they function as cooperative coactivators in androgen receptor-mediated transcription [6]. Che-1 was previously shown to interact with Rb and to affect its growth suppression activity by interfering with the Rb-mediated recruitment of histone deacetylase I to the promoters of E2F1-responsive genes [1,7]. However, Che-1 was also downregulated in several colon carcinomas and involved in growth arrest through induction of p21^Waf1 ^[8].

## Che-1 is involved in DNA damage responses

Cell cycle checkpoints are regulatory pathways that govern the order and timing of cell cycle transitions to ensure completion of one cellular event prior to commencement of another. The key regulators of the checkpoint pathways in the mammalian DNA damage response are ATM and ATR protein kinases [9]. Indeed, these proteins, once activated, phosphorylate directly or indirectly, via the checkpoint kinases Chk1 and Chk2, several important effectors such as p53, BRCA1, or NBS1 which are involved in cell cycle arrest, DNA repair, and apoptosis [10,11]. In particular, the p53 protein plays a critical role in the cellular response to DNA damage and other stresses by inhibiting proliferation or inducing apoptosis [12].

In a recent paper, the involvement of Che-1 in the cellular response to DNA damage was investigated [13]. In this study, Che-1 protein half-life was found to be regulated by the proteasome pathway and stabilized in response to physical and chemical genotoxic agents, an effect mediated by the kinases ATM and Chk2 which bind and phosphorylate Che-1 at four serine residues (Ser187 by ATM, and Ser141, Ser 474, and Ser508 by Chk2). Notably, substitution of these residues abolished Che-1 stabilization in response to DNA damage.

Since Che-1 is a RNA polymerase II binding protein, high-density Affimetrix microarrays were used to identify Che-1 target genes that might be relevant for the cellular response to genotoxic stress. Among the induced genes, the p53 oncosuppressor and several of its targets, e.g. cyclin G, p21, MDM2, and P53DINP1, were identified [13]. Consistent with these results, Che-1 depletion significantly decreased transcription of the p53 gene and other target genes both in basal conditions and upon DNA damage, supporting a role for Che-1 in their transcriptional regulation.

Chromatin immunoprecipitation experiments revealed that in response to DNA damage, Che-1 phosphorylation displaces it from E2F target genes and increases its occupancy at the *p21 *and *TP53 *promoters. From these findings it is possible to propose a model in which, in response to genotoxic stress, a molecular switch moves Che-1 from a cell cycle progression pathway to growth arrest of the cell. Bruno et al. [13] have shown that Che-1 interacts with the NF-kB subunit p65 in response to DNA damage, and that this interaction is mediated by Che-1 phosphorylation. Thus, post-translational modifications not only modulate Che-1 stability, but they may also mediate its interactions with transcription factors or co-factors and in this way regulate its presence on specific promoters. Che-1 was also found to be stabilized by phosphorylation during the G1/S transition, although the responsible kinase/s was not identified [7]. Therefore, several kinases may regulate Che-1 levels and interactions by different pathways depending upon specific cellular signals.

At the phenotypic level, Che-1 was found to be involved in the maintenance of the G2/M checkpoint in response to genotoxic stress, increasing the expression of p21^Waf1 ^and repression of CDC25C by p53 [13], whereas an involvement of Che-1 in the G1 checkpoint remains to be established. In addition, Che-1 inhibition strongly enhanced the cytotoxicity of anticancer drugs, thus suggesting Che-1 as a possible therapeutic target to increase the efficacy of DNA damaging drugs.

Based on these observations, it is reasonable to speculate that Che-1 may be a component of the anticancer barrier that protects cells from DNA damage or oncogenic stress [14,15] by inducing cellular senescence [16,17]. Consistent with this hypothesis, Che-1 contains a conserved motif (EExxxDDL) required by several important proteins involved in the DNA damage response, such as NSB1, Ku80 and ATRIP, for their interaction with ATM, DNA-PKcs, and ATR, respectively [18]. This motif is critical for kinase-mediated signaling events that trigger cell cycle checkpoints and DNA repair.

Recently, p53 isoforms were described [19], revealing the existence of an alternative p53 promoter and that expression of these isoforms can regulate p53 transcriptional activity. Thus, it is possible that Che-1 could also affect the activity of the p53 alternative promoter, regulating in this way the effect of p53 on growth arrest and apoptosis.

More recently, we demonstrated that, in response to apoptotic stimuli, HDM2/MDM2 protein negatively regulates Che-1 by promoting its ubiquitin-mediated degradation [20]. Che-1 was found to be a direct target of HDM2/MDM2 and that this interaction is increased following induction of apoptosis. The HDM2/MDM2-Che-1 interaction resulted in regulation by Pin1, which modifies the structure of Che-1, increasing its capacity to interact with HDM2/MDM2. Indeed, Pin1 bound Che-1 only upon Dox-mediated apoptosis, and siRNA-mediated Pin1 depletion strongly decreased HDM2/MDM2-Che-1 interactions, resulting in Che-1 stabilization [20].

In agreement with previous findings [4,7], Che-1 is confirmed to be an anti-apoptotic factor and that its degradation is required for executing the apoptotic program. Therefore, it possible to speculate that Pin1 is required for the apoptotic response to DNA damage not only by stabilizing p53 and p73 [21-23] but also by increasing Che-1 degradation.

## Che-1: additional roles in neuronal cell apoptosis and homeostasis

**Figure 1:**
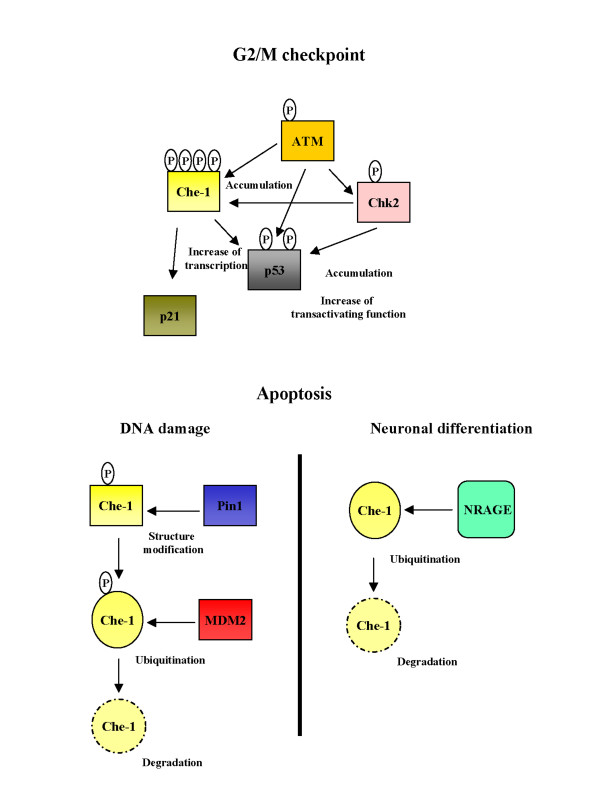
**Model to explain Che-1 modulation**. Non apoptotic genotoxic stresses induce Che-1 stabilization through its phosphorylation by ATM and Chk2. Stabilization of Che-1 produces an increase of p53 and p21 expression and contributes to arrest cell growth in response to DNA damage. Apoptotic DNA damage induces Che-1 Thr144 phosphorylation and Che-1/Pin1 interaction. Pin1 catalyzes Che-1 conformational changes, increasing Che-1/HDM2 interaction and in such way Che-1 degradation. In cortical neurons, NRAGE inhibits nuclear localization of Che-1, by sequestering it within the cytoplasmic compartment, and down-regulates endogenous Che-1 by targeting it for proteasome-dependent degradation.

Cell death programs are essential for embryonic development and maintenance of tissue homeostasis in multicellular organisms. Circumstantial evidence suggests that Che-1 is likely involved in apoptotic signaling in neural tissues in both normal and pathologic conditions. In particular, neuronal degeneration in Alzheimer's disease may be caused by extracellular accumulation of aggregated, neurotoxic amyloid β peptide 1–42. Che-1, in cortical neurons, exhibits anti-apoptotic activity, protecting cells from neuronal damage induced by amyloid β-peptide. Che-1 exerts this neuronal protection by interacting directly with the pro-apoptotic transcription factor Par-4 and inhibiting its ability to stimulate aberrant production of amyloid β peptide 1–42 [27,28]. In cerebellar granule neurons, Che-1 directly interacts with Tau, a microtubule-associated protein involved in the assembly and stabilization of the neuronal microtubule network that plays a crucial role in modulating neuronal morphogenesis, axonal shape, and transport. Tau undergoes a complex pattern of physiological and pathological post-translational changes, and it is the major component of the intraneuronal fibrillar lesions that characterize Alzheimer's disease and the neurodegenerative tauopathies [29]. Che-1/Tau association takes place in the cytoplasmic compartment of neuronal cells and it is finely modulated during the onset of neuronal apoptosis [30] (Fig 1).

Finally, Che-1 directly interacts with the neurotrophilin receptor-interacting MAGE homolog "NRAGE" [31]. NRAGE has been recently identified as a novel cell-death inducer involved in molecular events driving cells through apoptotic networks during neuronal development [32]. NRAGE inhibits Che-1 nuclear localization by sequestering it within the cytoplasmic compartment. Furthermore, while NRAGE overexpression downregulates endogenous Che-1 by targeting it for proteasome-dependent degradation, Che-1 acts as functional antagonist of NRAGE, since its overexpression completely reverts NRAGE-induced cell-death.

## Concluding remarks

Increasing evidence points at Che-1 as a critical actor in multiple molecular pathways. The significance of its role is growing because of its activities in transcriptional regulation, cell cycle control, DNA damage responses, and in the execution of cell death programs. Potential new roles for Che-1 in neuronal development, tissue homeostasis, and pathology underscore an even more complex molecular network. Finally, the emerging broad cytoprotective role of Che-1 identifies it as potential target for therapeutics.
